# 

**Published:** 1847-03

**Authors:** 


As this Journal has become the organ of the American Society of Dental Surgeons, and as its name has been somewhat modified, it may be considered as having entered upon a new period of its existence; and its former publishers congratulate themselves and the profession on its good fortune. Its perpetuity is now considered as settled. The profession will always need a periodical devoted to its interests, and who is better able to conduct it, than the only regularly organized and extensive association of dental surgeons in the world. We repeat, that we congratulate ourselves and our professional brethren on an event so auspicious to the science of dental surgery, as the formation of a national society and the transfer of the journal to that body. The work can now be regularly issued, whether its circulation be co-extensive with the profession or not. If the subscription should be limited, the issues can be made to correspond with the demand, so that the expense to the society may be kept wholly within its means.

The journal will be regularly issued on the first of December, March, June and September annually, at the enhanced price of five dollars per volume, payable in all cases in advance. The price of subscription may be paid to the treasurer of the society, Dr. C. A. Harris, Baltimore city, or to either of the editors, or any of the publishers, collaborators or acknowledged agents of the journal. Advance payment is required by a special resolution, passed at the last meeting of the society, and is necessary to protect it from pecuniary loss, a neglect of which has involved the publishers of the first volume in unexpected embarrassment, if not in ultimate loss.

